# Effects of Different Routes and Forms of Vitamin D Administration on Mesenteric Lymph Node CD4+ T Cell Polarization and Intestinal Injury in Obese Mice Complicated with Polymicrobial Sepsis

**DOI:** 10.3390/nu14173557

**Published:** 2022-08-29

**Authors:** Chiu-Li Yeh, Jin-Ming Wu, Kuen-Yuan Chen, Ming-Hsun Wu, Po-Jen Yang, Po-Chu Lee, Po-Da Chen, Sung-Ling Yeh, Ming-Tsan Lin

**Affiliations:** 1 School of Nutrition and Health Sciences, College of Nutrition, Taipei Medical University, Taipei 11031, Taiwan; 2 Research Center for Digestive Medicine, Taipei Medical University Hospital, Taipei 11031, Taiwan; 3 Department of Surgery, National Taiwan University Hospital and College of Medicine, National Taiwan University, Taipei 10002, Taiwan

**Keywords:** cholecalciferol, calcitriol, T helper cell, regulatory T cell, mucin, tight junction, AhR, interleukin-22

## Abstract

This study compared the efficacies of enteral cholecalciferol and/or intravenous (IV) calcitriol administration on mesenteric lymph node (MLN) cluster-of-differentiation-4-positive (CD4+) T cell distribution and intestinal barrier damage in obese mice complicated with sepsis. Mice were fed a high-fat diet for 16 weeks and then sepsis was induced by cecal ligation and puncture (CLP). Mice were divided into the following sepsis groups: without vitamin D (VD) (S); with oral cholecalciferol 1 day before CLP (G); with IV calcitriol 1 h after CLP (V); and with both cholecalciferol before and IV calcitriol after CLP (GV). All mice were sacrificed at 12 or 24 h after CLP. The findings show that the S group had a higher T helper (Th)17 percentage than the VD-treated groups at 12 h after CLP. The V group exhibited a higher Th1 percentage and Th1/Th2 ratio than the other groups at 24 h, whereas the V and GV groups had a lower Th17/regulatory T (Treg) ratio 12 h post-CLP in MLNs. In ileum tissues, the VD-treated groups had higher tight junction protein and cathelicidin levels, and higher mucin gene expression than the S group at 24 h post-CLP. Also, *aryl hydrocarbon receptor (AhR)* and its associated *cytochrome P450 1A1* and *interleukin 22* gene expressions were upregulated. In contrast, levels of lipid peroxides and inflammatory mediators in ileum tissues were lower in the groups with VD treatment after CLP. These results suggest that IV calcitriol seemed to have a more-pronounced effect on modulating the homeostasis of Th/Treg subsets in MLNs. Both oral cholecalciferol before and IV calcitriol after CLP promoted cathelicidin secretion, alleviated intestinal inflammation, and ameliorated the epithelial integrity in obese mice complicated with sepsis possibly via VD receptor and AhR signaling pathways.

## 1. Introduction

Sepsis, triggered by bacterial insults, is a heterogeneous syndrome that commonly occurs in intensive care units (ICUs) that leads to immune dysregulation, multiple organ failure, and even death in critically ill patients [1]. Obesity is a worldwide health issue that is closely associated with many metabolic disorders. Chronic low-grade systemic inflammation that results from obesity may lead to immune dysfunction and can adversely influence organ systems during the course of critical conditions [2,3]. Upon ICU admission, obese patients were found to have a higher risk of nosocomial infections that lead to sepsis [4,5].

Previous reports confirmed that gut-derived sepsis is closely associated with bacterial translocation [6,7]. The intestinal epithelium acts as barrier against pathogens within the lumen. When bacteria penetrate the epithelial layer, innate patrolling immune cells elicit inflammation that triggers adaptive immune responses by activating cluster-of-differentiation-4-positive (CD4+) T cells [8]. Sepsis-induced systemic inflammation is associated with primary damage to the intestinal barrier, which consequently promotes bacterial translocation [9,10]. Bacteria are first translocated to mesenteric lymph nodes (MLNs), then the spleen and internal organs, leading to bacteremia and sepsis progression [11,12]. A previous report showed that compared with normal mice, athymic mice were more susceptible to a variety of bacterial infections. This phenomenon suggests that the T-lymphocyte-mediated immune response plays crucial roles in conserving the intestinal barrier and inhibiting bacterial translocation [13]. The CD4+ T cell is one of the T lymphocyte subsets that modulates adaptive and humoral immunity following an infection [14]. Orchestrating the balance among CD4+ T cell subpopulations and preventing damage to the intestinal barrier are important strategies for managing sepsis.

Vitamin D (VD) is a hormonal nutrient that has modulatory effects on immune responses [15,16]. Previous studies found that VD modulates the differentiation of different subsets of T lymphocytes that may exert protection against diseases [17,18]. Also, VD was reported to play pivotal roles in stabilizing intestinal epithelial junctions, and balancing the gut microbiota and intestinal immunity, thus maintaining homeostasis of the gut barrier [8]. Low VD levels were inversely linked to recurrence and disease severity of inflammatory bowel disease (IBD) [19,20], and were also a risk factor for critically ill populations [21,22]. The canonical pathway of VD exerts its physiological functions by interacting with VD receptor (VDR) [23]. Recently, a noncanonical pathway involving the aryl hydrocarbon receptor (AhR) was found to be closely associated with the anti-inflammatory, antioxidant and immunomodulatory properties of VD [24,25]. In parallel, the AhR signaling pathway maintains the homeostasis of intestinal immunity and prevents barrier dysfunction [26,27]. The correlation of VD/AhR and intestinal integrity during sepsis is worthy of investigation.

Several clinical studies investigated the impact of VD supplementation on biochemical alterations and clinical outcomes in critically ill patients. Most clinical studies provided mega-dose enteral cholecalciferol [28,29,30]. Calcitriol (1,25(OH)2D) is the active form of VD. Commercial calcitriol is commonly used to treat mineral disorders and osteodystrophy in chronic kidney disease by intravenous (IV) formulations [31]. Clinical trials investigating calcitriol treatment in critical illnesses are rare. A study by Leaf et al. revealed that calcitriol administration had no effect on clinical outcomes but increased leukocyte gene expression of cathelicidin in septic patients [32]. An animal study reported that a subcutaneous injection of calcitriol improved the intestinal barrier integrity and its function in rats with liver cirrhosis [33]. Some studies also reported that cholecalciferol or calcitriol administration to rodents ameliorated experimental IBD [20,34]. However, very few studies have investigated cholecalciferol or calcitriol treatment of intestinal injuries in sepsis. As we know, there is no study that has compared the efficacies between cholecalciferol and calcitriol in sepsis-induced CD4+ T cell changes and associated intestinal inflammation and damage. In this study, diet-induced obesity in mice was established, and clinically relevant dosages of oral cholecalciferol and/or IV calcitriol were administered to investigate the impact of different forms and routes of VD on MLN CD4+ T cell polarization and intestinal barrier damage in obesity complicated with sepsis.

## 2. Materials and Methods

### 2.1. Animal Preparations

Five-week-old male C57BL/6 mice (weighing 17~18 g) were used in this study. All animals were maintained in a humidity- (55% ± 2%) and temperature (22 ± 2 °C)-controlled room with a 12-h light/dark cycle in the Laboratory Animal Center of Taipei Medical University (TMU; Taipei, Taiwan). Mice were fed a rodent chow diet (Purina no. 5001, Fort Worth, TX, USA) during the accommodation period. This study was approved by the Institutional Animal Care and Use Committee of TMU (LAC-2021-0199).

### 2.2. Experimental Procedures

Eighty mice were fed a high-fat diet (HFD) for 16 weeks to induce obesity. The diet contained 60% of calories as fat. Diet compositions are presented in Table 1, and they were supplied by a commercial company (Research Diets, New Brunswick, NJ, USA). Sepsis was induced in obese mice with comparable body weights (BWs) by cecal ligation and puncture (CLP), and then the mice were divided into four groups as follows: group S, sepsis without VD but an equal volume of oral gavage of coconut oil 1 day before, and an IV saline injection via a tail vein 1 h after CLP as provided in groups G and V; group G, oral cholecalciferol (2000 IU/mice) (LiquiD P&B., ULONG Pharmaceutical, Taipei, Taiwan) gavage 1 day before and an IV saline injection 1 h after CLP; group V, oral gavage of coconut oil 1 day before and IV calcitriol (410 ng/kg BW) (Cacare injection, Nang Kuang Pharmaceutical, Taipei, Taiwan) 1 h after CLP; and group GV, with both cholecalciferol before and IV calcitriol after CLP. The CLP procedures were described in detail in our previous study [35]. After the operation, sterile saline was injected subcutaneously for rehydration. All mice were given rodent chow diet and water freely during the period of recovery. Mice in the experimental groups were sacrificed at 12 or 24 h after the operation (n = 8 in each group at each time point). The dosages of cholecalciferol (3 × 10^5^ IU daily) and calcitriol (2 µg/day) we chose were referenced from clinical trials, and these levels were considered to have benefits in critically ill patients [32,36]. The dose conversion between mice and humans was calculated according to practice guidelines for animal studies [37]. All mice were anesthetized with Zoletil (25 mg/kg BW) and Rompun (10 mg/kg BW) injected intraperitoneally and euthanized by cardiac puncture at the end of the experiment. The peritoneum was opened and peritoneal lavage fluid (PLF) was obtained by irrigating with saline. MLNs and the distal end of ileum tissues were collected. Fresh MLNs were used to analyze the distribution of CD4+ T cell subsets. Ileum tissues were frozen at −80 °C for further measurements.

### 2.3. Inflammatory Mediator Concentrations in PLF

Interleukin (IL)-1β, IL-6, tumor necrosis factor (TNF)-α, and monocyte chemoattractant protein (MCP)-1 were measured by enzyme-linked immunosorbent assays (ELISAs) (R&D Systems, Minneapolis, MN, USA). Levels of these parameters were adjusted by the protein concentration in the PLF. Protein concentrations were measured with a commercial assay kit (Bio-Rad, Hercules, CA, USA).

### 2.4. Distribution of CD4+ T Cell Subpopulations in MLNs

MLNs were freshly prepared and single-cell suspensions were obtained. The pellets centrifuged from the original suspensions were re-suspended in cell staining buffer (Biolegend, San Diego, CA, USA) and were used to analyze T helper (Th) and regulatory T (Treg) cell subsets. All procedures were described in detail in a previous experiment performed by our laboratory [38]. Extracellular staining antibodies (Abs), including CD3-Pacific blue (PB) (17A2, Biolegend, San Diego, CA, USA) and CD4-peridinin-chlorophyll-protein (PerCP) (GK1.5, Biolegend, San Diego, CA, USA) were applied to measure the distribution of Th cell populations. To analyze subpopulations of Th cells, specific Abs were used for intracellular cytokine staining: interferon (IFN)-γ-allophycocyanin (APC) (XMG1.2, Biolegend, San Diego, CA, USA), IL-4-phycoerythrin (PE) (11B11, Biolegend, San Diego, CA, USA), and IL-17A-fluorescein (FITC) (TC11-18H10.1, Biolegend, San Diego, CA, USA). Phenotypes of Th cells are presented as percentages of Th-associated cytokine-expressing cells among CD4+ T cells: CD3+/CD4+/IFN-γ+, CD3+/CD4+/IL-4+, and CD3+/CD4+/IL-17A+ cells represent Th1, Th2, and Th17 cells, respectively. To determine Treg cells, CD4-APC (GK1.5, Biolegend, San Diego, CA, USA) and CD25- PE (3C7, Biolegend, San Diego, CA, USA) Abs were used. Foxp3-Alexa Flour 488 Abs (MF-14, Biolegend, San Diego, CA, USA) were applied for intracellular staining. Treg cells are presented as a percentage of CD4+/CD25+/Foxp3+ cells among CD4+/CD25+ cells. Each single-color compensation control was settled with UltraComp eBeads™ Plus Compensation Beads (Invitrogen, Carlsbad, CA, USA). Fluorescent samples were analyzed with an Attune™ NxT Acoustic Focusing Cytometer (Invitrogen, Carlsbad, CA, USA). Lymphocytes were gated with characteristics of a small size and few granules.

### 2.5. Tight Junction (TJ) Protein Levels in Ileum Tissues

Ileum tissues, 1 cm proximal to the cecum, were used and homogenized in Tissue Protein Extraction Reagent (T-PER™, Thermo Fisher Scientific, Vilnius, Lithuania) with a protease and phosphatase inhibitor (Thermo Fisher Scientific, Vilnius, Lithuania). Supernatants were collected after centrifugation. Zona occludens (ZO)-1, occluding, and claudin were detected with Mouse TJP1 (Tight junction protein 1), Mouse OCLN (Occludin), and Mouse Cldn1 (Claudin-1) ELISA kits (FineTest, Wuhan, Hubei, China). Protein concentrations were measured (Bio-Rad, Hercules, CA, USA), and the data are expressed as pg/mg protein.

### 2.6. Concentrations of Cathelicidin in Ileum Tissues

Homogenized ileum tissues were obtained as mentioned above. After centrifugation, supernatants were used to quantify cathelicidin levels with a Mouse Cathelicidin Antimicrobial Peptide (CAMP) ELISA kit (ABclonal, Woburn, MA, USA). Samples were read at an absorbance of 450 nm and corrected at 570 or 630 nm with a spectrophotometer [35]. Concentrations are expressed as ng/mg of tissue protein and a commercial assay kit (Bio-Rad, Hercules, CA, USA) was used to measure protein concentrations.

### 2.7. Messenger (m)RNA Extraction and Analysis of a Real-Time Reverse-Transcription (RT) Quantitative Polymerase Chain Reaction (qPCR)

Ileum tissues were homogenized. Total RNA was isolated and RNA pellets were dissolved in RNase-free water and stored at −80 °C for further analysis. The reagents and instrument used, analyzing conditions and all procedures were described in detail in our previous experiments [35,38]. The genes measured included *VD receptor (VDR), cytochrome P450 27B1 (CYP27B1), mucin 2 (Muc2),* and *trefoil factor 3 (Tff3). AhR* and its associated *CYP1A1, interleukin (IL)-22,* and *IL-22 receptor (IL-22R*). Primers used are presented in Table 2. All primers were provided by Mission Biotech (Taipei, Taiwan) based on deposited cDNA sequences (GenBank database, NCBI). Expression levels of genes were quantified in duplicate by a real-time RT-PCR. The relative mRNA expression was calculated by cycle threshold (CT) values and normalized to mouse *glyceraldehyde 3-phosphate dehydrogenase (GAPDH*).

### 2.8. Analysis of Thiobarbituric-Acid-Reactive Substances (TBARSs) in the Ileum

Tissues were homogenized with reagent as mentioned above. Supernatants were used to analyze TBARSs using a commercial TBARs assay kit (Cayman, Ann Arbor, MI, USA) as described previously [38]. TBARSs are formed when malondialdehyde (MDA) reacts with thiobarbituric acid. MDA is the representative end-product during the decomposition of lipid peroxidation. The concentrations of TBARs in the biological samples are considered as indicators of lipid peroxidation and oxidative stress [39]. TBARSs levels are presented as μmole/g protein. Protein concentrations were analyzed by commercial assay kit (Bio-Rad, Hercules, CA, USA).

### 2.9. Statistical Analysis

Data are expressed as the mean ± standard error of the mean (SEM). Statistical analyses were performed with GraphPad Prism 5.0 software (La Jolla, CA, USA). A one-way analysis of variance (ANOVA) followed by Tukey’s post hoc test was used to analyze differences among the sepsis groups at the same time point. A *p* value of <0.05 was considered statistically significant.

## 3. Results

### 3.1. PLF Inflammatory Mediator Concentrations

**Figure 1:**
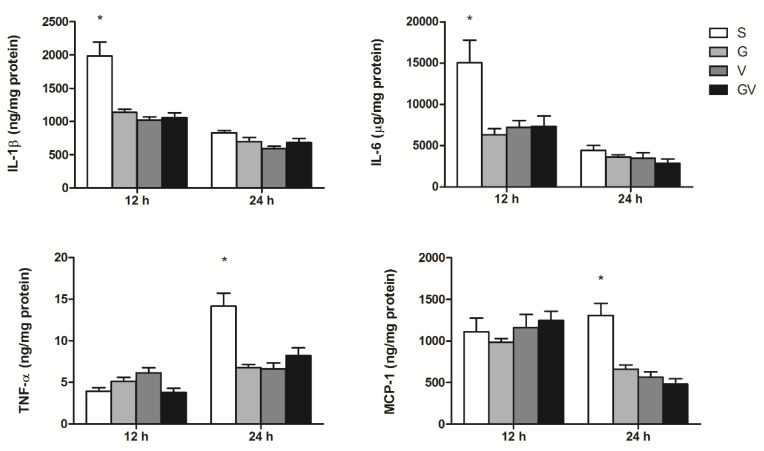
Concentrations of inflammatory mediators in peritoneal lavage fluid after cecal ligation and puncture (CLP) at two time points. IL-1β, interleukin-1β; IL-6, interleukin-6; TNF, tumor necrosis factor; MCP, macrophage chemoattractant protein; S, sepsis group without vitamin D (VD) before or after CLP; G, sepsis group with cholecalciferol gavage before CLP; V, sepsis group with an intravenous calcitriol injection after CLP; GV, sepsis group with both cholecalciferol before and intravenous calcitriol after CLP (*n* = 8 in each group). Data are presented as the mean ± standard error of the mean (SEM). Differences among groups at the same time point were analyzed by a one-way analysis of variance using Tukey’s post hoc test. * Significantly differs from the other groups at the same time point (*p* < 0.05).

The S group had higher IL-1β and IL-6 levels at 12 h, and TNF-α and MCP-1 at 24 h after CLP than those expressed in the VD-treated groups. There were no differences in IL-1β, IL-6, TNF-α, or MCP-1 levels among the VD-treated groups at either time point (Figure 1).

### 3.2. Distribution of CD4 T Cell Subpopulations in MLNs

**Figure 2:**
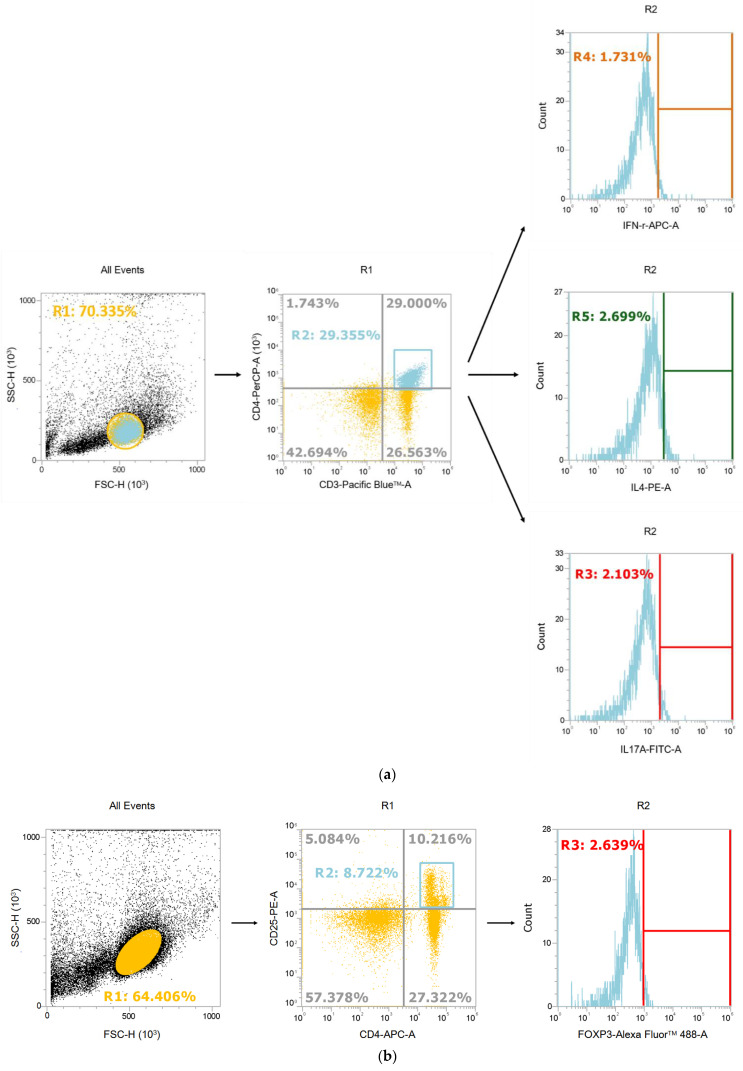

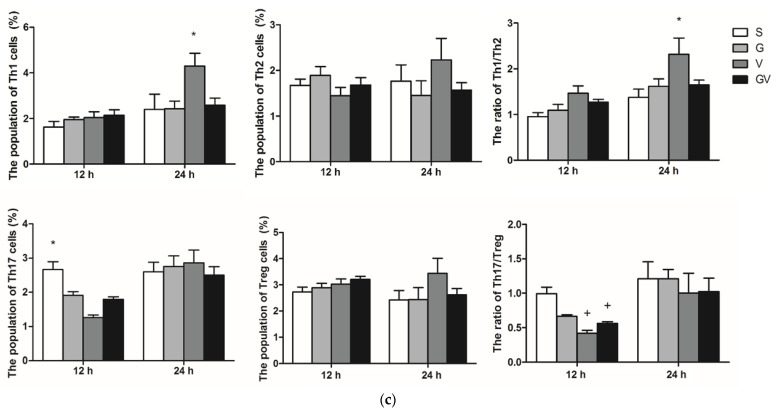
Representative flow cytometry plots and percentages of T helper (Th) and regulatory T (Treg) cell subpopulations in mesenteric lymph nodes (MLNs) after cecal ligation and puncture at two time points. (**a**) Gating strategy for Th type 1 (Th1), Th2, and Th17. Lymphocytes were first identified based on Side Scatter (SSC) and Forward Scatter (FSC) characteristics. Cluster-of-differentiation-4-positive (CD4+) lymphocytes were gated to analyze the percentages of interferon (IFN)-γ-expressing, interleukin (IL)-4-expressing, and IL-17-expressing CD4 T cells. (**b**) Gating strategy for Treg cells. Forkhead box p3 (Foxp3)-expressing CD4+ lymphocytes were gated to identify the percentages of Treg cells. (**c**) Percentages of Th, Th2, Th17, and Treg cell subpopulations and the ratios of Th1/Th2 and Th17/Treg. The groups are described in the legend of Figure 1 (*n* = 8 in each group). Data are presented as the mean ± SEM. Differences among groups at the same time point were analyzed by a one-way analysis of variance using Tukey’s post hoc test. * Significantly differs from the other groups at the same time point. ^+^ Significantly differs from the S and G groups at the same time point (*p* < 0.05).

Representative flow cytometry plots are shown to illustrate the typical gating strategy for Th subsets (Figure 2a) and Treg cells (Figure 2b). The distribution of the CD4 T cell subsets showed that the S group had a significantly higher Th17 percentage than the VD-treated groups. No differences in Th1, Th2, or Treg percentages were found among the experimental groups at 12 h after CLP. At 24 h post-CLP, there were no differences in Th2, Th17, or Treg percentages among the four groups. However, the V group exhibited a higher Th1 percentage and Th1/Th2 ratio than the other three groups. Also, the V and GV groups showed lower Th17/Treg ratios than did the S and G groups (Figure 2c).

### 3.3. Inflammatory Mediator Concentrations in Intestinal Tissues

**Figure 3:**
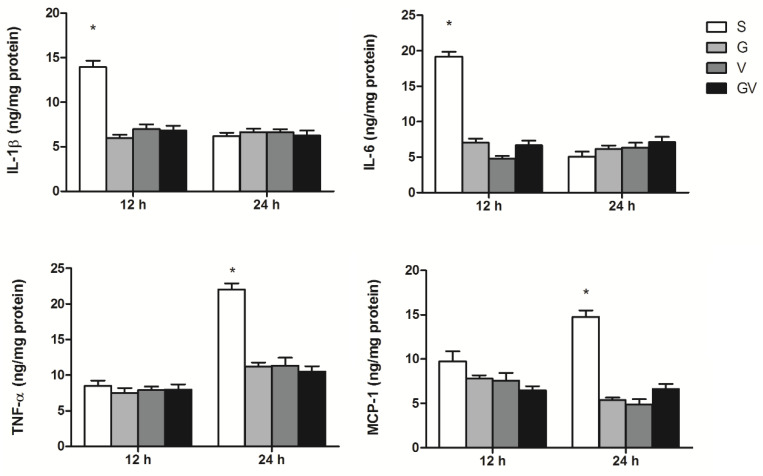
Concentrations of inflammatory mediators in ileum tissues after cecal ligation and puncture at two time points. IL, interleukin; TNF, tumor necrosis factor; MCP, macrophage chemoattractant protein. The groups are described in the legend of Figure 1 (*n* = 8 in each group). Data are presented as the mean ± SEM. Differences among groups at the same time point were analyzed by a one-way analysis of variance using Tukey’s post hoc test. * Significantly differs from the other groups at the same time point (*p* < 0.05).

Concentrations of IL-1β and IL-6 at 12 h, and TNF-α and MCP-1 at 24 h post-CLP were significantly higher in the S group than those in the VD-treated groups. These inflammatory mediators did not differ among the VD-treated groups at either time point (Figure 3).

### 3.4. VDR and CYP27B1 Gene Expressions in the Ileum

**Figure 4:**
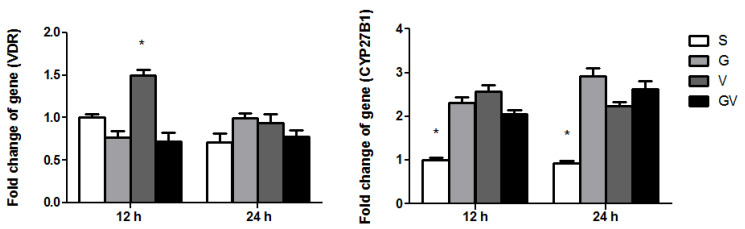
Messenger (m)RNA expression levels of the vitamin D receptor (VDR) and cytochrome p450 27B1 (CYP27B1) genes in ileum tissues after cecal ligation and puncture at two time points. mRNA changes were quantitated and analyzed by real-time PCR and were calculated using the comparative CT (2^−ΔΔCt^) method. mRNA expression levels in the S group were used as a calibrator. Data are presented as the mean ± standard error of the mean (SEM). *n* = 8 for each group. The groups are described in the legend of Figure 1. Differences among groups at the same time point were analyzed by a one-way analysis of variance using Tukey’s post hoc test. * Significantly differs from the other groups at the same time point (*p* < 0.05).

The V group had the highest *VDR* gene expression at 12 h after CLP. There were no differences in *VDR* expression among the experimental groups at 24 h post-CLP. The VD-treated groups had higher *CYP27B1* mRNA expression levels than the S group after CLP (Figure 4).

### 3.5. Cathelicidin Levels in the Ileum

**Figure 5:**
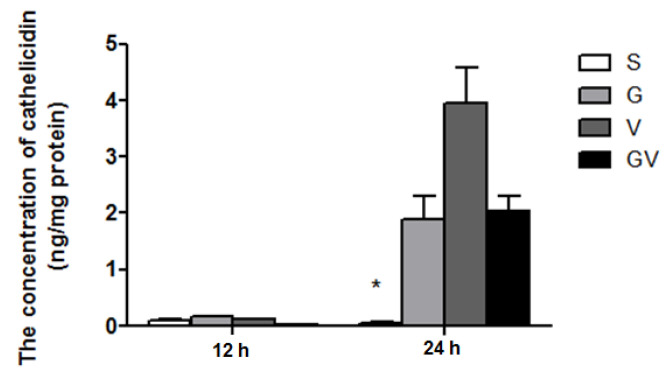
Cathelicidin concentrations in ileum tissues after cecal ligation and puncture at two time points. Data are presented as the mean ± standard error of the mean (SEM). *n* = 8 for each group. The groups are described in the legend of Figure 1. Differences among groups at the same time point were analyzed by a one-way analysis of variance using Tukey’s post hoc test. * Significantly differs from the other groups at the same time point (*p* < 0.05).

Levels of cathelicidin did not differ among the experimental groups at 12 h after CLP. At 24 h, cathelicidin levels increased in the VD-treated groups, which were significantly higher than the S group (Figure 5).

### 3.6. AhR-Associated Gene Expression in Ileum Tissues

**Figure 6:**
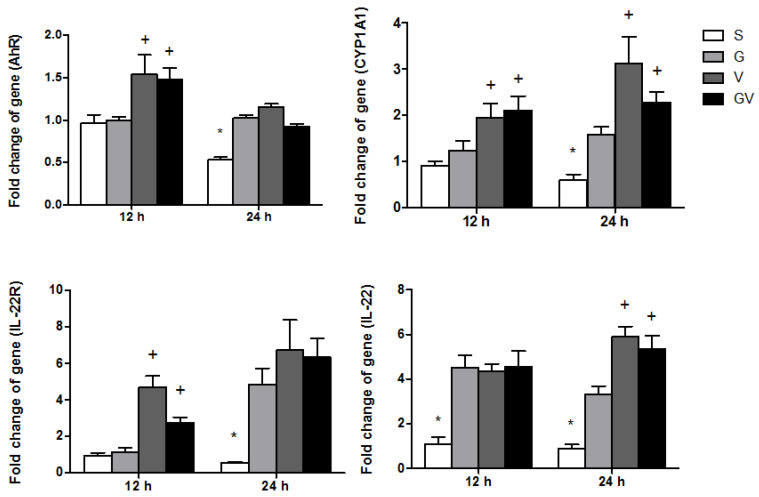
Aryl hydrocarbon receptor (AhR), cytochrome P450 1A1 (CYP1A1), interleukin (IL)-22, and IL-22 receptor (IL-22R) mRNA levels in ileum tissues after cecal ligation and puncture at two time points. mRNA changes were quantitated and analyzed by real-time PCR and were calculated by the comparative CT (2^−ΔΔCt^) method. mRNA expression levels in the S group were used as a calibrator. The groups are described in the legend of Figure 1. Data are shown as the mean ± SEM (*n* = 8 for each group). Differences among groups at the same time point were analyzed by a one-way analysis of variance using Tukey’s post hoc test. * Significantly differs from the other groups at the same time point. ^+^ Significantly differs from the S and G groups at the same time point (*p* < 0.05).

The gene expression levels of *AhR, CYP1A1,* and *IL-22R* in the V and GV groups were significantly higher than the S and G groups, whereas all the VD-treated groups had higher *IL-22* expression than the S group at 12 h after CLP. At the time point at 24 h, the groups with VD treatment showed higher *AhR, CYP1A1, IL-22,* and *IL-22R* expression levels than those expressed in the S group. Among the three VD-treated groups, V and GV groups exhibited higher *CYP1A1* and *IL-22* expression than the G group (Figure 6).

### 3.7. Expressions of TJ Protein Levels and Mucosal Repair Genes in the Ileum

**Figure 7:**
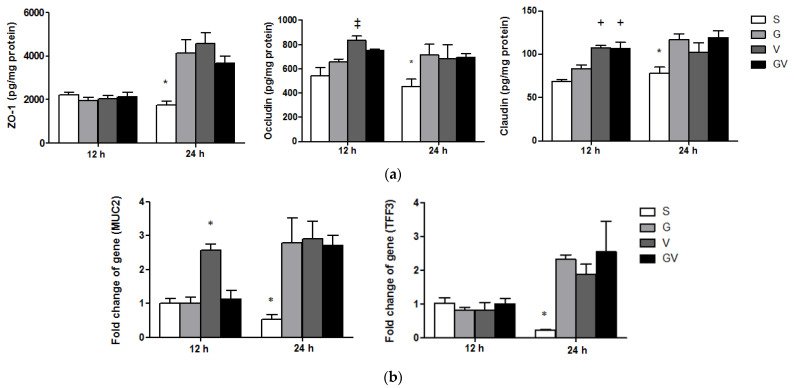
(**a**) Tight junction protein levels and (**b**) mRNA levels of mucins in ileum tissues after cecal ligation and puncture at two time points. ZO-1, Zona occludens-1; Muc2, mucin 2; Tff3, trefoil factor 3. mRNA changes were quantitated and analyzed by real-time PCR and were calculated by the comparative CT (2^−ΔΔCt^) method. mRNA expression levels in the S group were used as a calibrator. The groups are described in the legend of Figure 1. Data are shown as the mean ± SEM (n = 8 for each group). Differences among groups at the same time point were analyzed by a one-way analysis of variance using Tukey’s post hoc test. * Significantly differs from the other groups at the same time point. ^+^ Significantly differs from the S and G groups at the same time point. ^‡^ Significantly differs from the S group at the same time point (*p* < 0.05).

There were no differences in ZO-1 levels among the experimental groups, however, the concentrations of occludin in the V group were higher than the S group, and claudin in the V and GV groups was higher than in the S and G groups at 12 h post-CLP. The VD-treated groups had higher ZO-1, occludin, and claudin levels than did the S group, but no differences were observed among the three VD-treated groups at 24 h after CLP (Figure 7a). *MUC2* expression in the V group was the highest; however, no differences in *TFF3* expression was observed among the four experimental groups at 12 h after CLP. At 24 h post-CLP, *MUC2* and *TFF3* gene expressions were significantly higher in the VD-treated groups than those in the S group (Figure 7b).

### 3.8. Lipid Peroxide Concentrations in the Ileum

**Figure 8:**
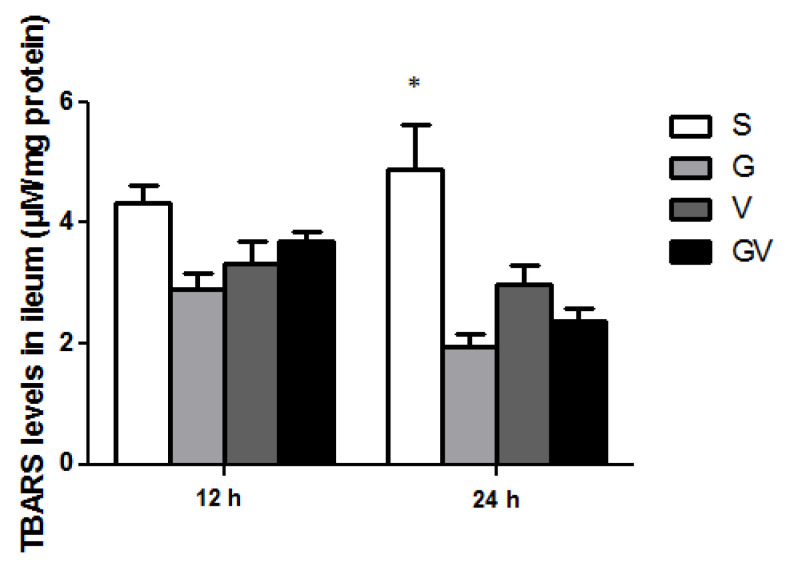
Thiobarbituric-acid-reactive substance (TBARS) levels in ileum tissues after cecal ligation and puncture at two time points. The groups are described in the legend of Figure 1 (n = 8 in each group). Values are expressed as the mean ± SEM. Differences among groups at the same time point were analyzed by a one-way analysis of variance using Tukey’s post hoc test. * Significantly differs from the other groups at the same time point (*p* < 0.05).

No differences in TBARS levels were observed among the four experimental groups at 12 h after CLP. However, the VD-treated groups exhibited lower TBARS levels than the S group at 24 h post-CLP (Figure 8).

## 4. Discussion

In this study, we did not include an obesity group without sepsis, because our previous study confirmed that HFD-induced obesity results in excessive body fat accumulation and proinflammatory mediator production. In parallel, sepsis further enhances more pronounced inflammation in obesity [38]. Since there are close links between VD/VDR and VD/AhR signaling with tissue barriers and epithelial integrity [8,40], this study compared the efficacies of different forms of VD with clinically relevant dosages on MLN CD4+ T cell polarization and their association with intestinal inflammation and injury in a critical condition of obesity complicated with sepsis. Cholecalciferol is the form of VD that is mostly recognized in clinical guidelines for managing a VD deficiency [41]. Ingested cholecalciferol is metabolized by VD-25-hydroxylase in the liver to 25(OH)D, and further hydrolyzed by 25-hydroxyvitamin-D-1α-hydroxylase (CYP27B1) to calcitriol in the kidney and other organs. In this study, cholecalciferol was provided before sepsis because critical illness may cause gastrointestinal function to deteriorate [42]. Calcitriol is a readily used form for IV injections. We administered calcitriol after CLP, which may have compensated for the impaired ability of VD conversion to its active form during sepsis. The findings of this study revealed that both cholecalciferol before and calcitriol after sepsis alleviated intestinal inflammation and barrier injuries in an obese animal model.

MLNs are an important tissue responsible for T cell activation in the intestines [43]. In this study, CD4+ T cell subsets including Th and Treg percentages were analyzed in MLNs. According to distinct cytokine secretions and effector functions, Th cells are classified as Th1, Th2, Th17, and Th22 in response to infections. Th1 cells promote cell-mediated immunity, whereas Th2 cells enhance humoral immunity [44]. Cytokines secreted by Th17 cells were found to be associated with tissue inflammation [45]. Th22 cells produce IL-22 that may attenuate inflammatory responses during diseases [46]. Treg cells have the opposite actions to Th17, thus suppressing excessive T cell responses during inflammatory conditions [47]. The balance among the subpopulations of Th and Treg cells as well as cytokines derived from these CD4+ T cells play crucial roles in the persistence and progression of sepsis [48]. In this study, we found that calcitriol treatment alone led to a higher Th1 distribution and Th1/Th2 ratio in the late phase. Also, the V and GV groups had lower Th17/Treg ratios than the G and S groups in the earlier stage of sepsis. A former study found that sepsis patients had lower Th1/Th2 ratios than the non-septic controls [49]. A clinical study also showed that non-survivors had higher Th17/Treg ratios than did surviving septic patients. CD4+ T cells that shifted towards Th2- and Th17-type responses produced poorer outcomes in septic patients [50]. The Th2 subtype is susceptible to infection [51], and the Th17-mediated immune responses enhance systemic inflammation during sepsis [52]. The higher Th1/Th2 ratio and lower Th17/Treg ratio observed in the calcitriol-treated groups suggest that a more-balanced CD4+ T cell distribution was obtained. Since this finding was not observed in the G group, the favorable effect of modulating the distribution of CD4 T cell subsets may have been derived from calcitriol treatment. Calcitriol was found to influence adaptive immunity by modulating the polarization of Th and Treg cells in disease states [18]. Our previous report also showed that calcitriol lowered the distribution of Th2 and Th17 during the course of sepsis [38]. In this study, we found the expression of Th1 increased, but Treg did not change in the calcitriol-treated group. These findings were inconsistent with former studies that showed that calcitriol inhibits the Th1 response and promotes the differentiation of Treg cells in a mouse model of non-obese diabetes [53] and has favorable effects on attenuating the severity of autoimmune diseases [54,55]. Since the characteristics and the distribution of T lymphocyte subsets in autoimmune diseases are totally different from polymicrobial sepsis, the impacts of calcitriol on the polarization of CD4+ T cell subpopulations may differ. VDR is a nuclear receptor that is highly expressed in the intestines and by immune cells [18,23]. Directly activated by calcitriol, the VDR regulates diverse genes to exert its influences on immune functions [56]. In this study, we noted that the group with calcitriol treatment had higher VDR expression in the intestines. Calcitriol interacts with the VDR to possibly modulate different subgroups of CD4+ T lymphocytes in MLNs. The bioavailability of calcitriol immediately after sepsis may have greater benefits than antecedent cholecalciferol administration in sepsis-induced T cell dysregulation. Cathelicidin is an antimicrobial peptide that confers protection against bacterial infection [57]. Calcitriol is a direct inducer of cathelicidin expression [58]. The higher cathelicidin produced by all of the VD-treated groups suggests that cholecalciferol had an equivalent ability to stimulate innate immunity. A study performed by Ho et al. also found that pretreatment with cholecalciferol enhanced ileal cathelicidin expression in a mouse CLP model [59].

In this study, several inflammatory mediators and indicators were analyzed. IL-1β, IL-6, and TNF-α are inflammatory cytokines. MCP-1 is a chemokine that modulates the infiltration and migration of monocytes/macrophages [60]. We found that these mediators in the intestines and PLF had decreased at either 12 or 24 h after sepsis in all of the VD-treated groups. These findings suggest that both cholecalciferol and/or calcitriol administration attenuate intestinal inflammation in obesity concurrent with sepsis. Previous studies found that in mice with dietary cholecalciferol administration, colonic inflammation decreased and IBD was alleviated [61,62]. Also, calcitriol downregulates toll-like-receptor-4-mediated inflammation by inhibiting nuclear factor-κB activation in monocytes/macrophages [63].

AhR is a nuclear transcription factor that is essential for regulating several signaling pathways. Hydroxyderivaties of cholecalciferol and calcitriol can interact with AhR, thus enhancing the activation of AhR signaling [25]. In this study, we analyzed several genes related to the AhR pathway. Cyp1A1 is one of the target genes that is modulated by AhR. CYP1A1 expression is used to evaluate the activity of AhR [64]. Also, IL-22 is the downstream cytokine in the AhR signaling [65]. IL-22 binds to the IL-22R expressed on the epithelial cells of the organs such as the lung, skin, and gut and exerts its function in preserving the integrity of the barrier tissues [66]. The upregulation of ileal AhR-associated gene expression after sepsis suggests AhR signaling is activated when cholecalciferol and/or calcitriol were administered.

The intestinal epithelium is comprised of a single layer of cells that segregates commensal microorganisms from host tissues to maintain the homeostasis of the intestinal environment. Spaces between epithelial cells are tightly integrated by tight junction (TJ) proteins that regulate the permeability of the intestinal epithelium. Destruction of TJs results in barrier dysfunction that is associated with adverse outcomes during sepsis [67]. Occludin and claudin are major transmembrane TJ proteins. ZO-1 is an adaptor protein that interacts with other junctional components in maintaining optimal barrier functions [68,69]. Meanwhile, a layer of mucus lining the epithelium forms a protective physical barrier to resist initial injury and sustain the epithelial integrity. This layer is constituted by mucins. Muc2 is the major secreted mucin found in the intestinal mucus [70]. Trefoils are small peptides that play crucial roles in mucosal protection and repair. Tff3, one factor of the trefoil peptide family, is highly expressed in the intestinal mucosa that is secreted by goblet cells [71]. In this study, we found that sepsis groups with either cholecalciferol or calcitriol administration had higher TJ and mucin expressions, especially at the late phase of sepsis. On the other hand, the CYP27B1 expression in the intestines was comparable between cholecalciferol- and calcitriol-treated groups, which suggests that oral cholecalciferol administration before sepsis can be well utilized and metabolized in the intestines to exert its biological functions similarly to calcitriol. These findings suggest that both forms of VD can act via the canonical pathway by VDR or noncanonical pathway involving AhR to attenuate intestinal inflammation and improve epithelial barrier integrity in sepsis. However, the molecular mechanisms require further investigation.

A study performed by Ho et al. found that cholecalciferol administration after CLP adversely affects 7-day mortality and associated disease symptoms [59]. Because sepsis is associated with structural alterations in the small intestine and impaired metabolic functions of the gut, oral VD administration might not be advisable after sepsis. IV calcitriol treatment after sepsis would be more clinically relevant for this critical condition. Compared to the mega-dose of cholecalciferol, the dosage of calcitriol is relatively low and can readily be used without increasing the metabolic load during the course of sepsis.

## 5. Conclusions

In summary, this is the first study to compare the efficacies of clinically relevant doses, administration routes, and timing of cholecalciferol and calcitriol treatments on MLN CD4+ T cell polarization and associated intestinal injury in obesity concurrent with sepsis. The findings revealed that calcitriol seemed to have a more pronounced effect on modulating the homeostasis of Th/Treg subsets in MLNs. Both oral cholecalciferol before and IV calcitriol after CLP promoted cathelicidin secretion, upregulated AhR-associated gene expression, alleviated intestinal inflammation, and improved the epithelial integrity possibly via the VD/VDR and VD/AhR signaling pathways. The findings of this research may provide a new understanding for choosing the route and applicable dosage for critically obese patients. Prophylactic oral cholecalciferol may preferentially be provided to patients at risk of sepsis whereas IV calcitriol can readily be used after the onset of sepsis.

## Figures and Tables

**Table 1:** Composition of the high-fat diet.

Ingredient (g)	High-Fat Diet
Casein, lactic	258.45
L-Cystine	3.88
Corn starch	-
Maltodextrin	161.53
Sucrose	94.08
Cellulose	64.61
Lard	316.60
Soybean oil	32.31
Mineral mix ^1^	64.61
Choline bitartrate	2.58
Vitamin mix ^2^	1.29
Dye	0.06
Total	1000
Protein/Fat/Carbohydrates (%)	20/60/20
Energy density (kcal/g)	5.21

^1^ The composition of the mineral mixture is listed as follows (g/1000 g): potassium citrate, 330; calcium phosphate, 260; calcium carbonate, 110; sodium chloride, 51.8; magnesium sulfate, 51.52; magnesium oxide, 8.38; ferric citrate 4.2; manganese carbohydrate hydrate, 2.45; zinc carbonate, 1.12; chromium potassium sulfate, 0.39; copper carbonate, 0.21; ammonium molybdate tetrahydrate, 0.06; sodium fluoride, 0.04; sodium selenite, 0.01; potassium iodate, 0.01. ^2^ The composition of the vitamin mixture is listed as follows (g/100 g): vitamin E acetate, 10; niacin, 3; biotin (1%), 2; pantothenic acid, 1.6; vitamin D3, 1; vitamin B12, 1; vitamin A acetate, 0.8; pyridoxine hydrochloride (HCL), 0.7; riboflavin, 0.6; thiamine HCL, 0.6; folic acid, 0.2; menadione sodium bisulfite, 0.08.

**Table 2:** Sequences of oligonucleotide primers used for PCR amplification.

Gene Name	Primer Sequence (5′ → 3′)	Accession No.
*AhR*	F: GGCTTTCAGCAGTCTGATGTC	AF405563.1
	R: CATGAAAGAAGCGTTCTCTGG	
*CYP1A1*	F: CAATGAGTTTGGGGAGGTTACTG	NM_009992.4
	R: CCCTTCTCAAATGTCCTGTAGTG	
*CYP27B1*	F: GGTTCTCCGGAGCTTGTCTG	NM_010009.2
	R: AAACTGTGCGAAGTGTCCCA	
*IL-22*	F: TTTCCTGACCAAACTCAGCA	XM_006513865.4
	R: TCTGGATGTTCTGGTCGTCA	
*IL-22Rα*	F: CACACCGGTCCTCTCGGAAG	NM_178257.2
	R: GGCACTTTCCTTGGACAATATCGG	
*Muc2*	F: ATGCCCACCTCCTCAAAGAC	BC039285.1
	R: GTAGTTTCCGTTGGAACAGTGAA	
*Tff3*	F: TAATGCTGTTGGTGGTCCTG	NM_011575.2
	R: CAGCCACGGTTGTTACACTG	
*VDR*	F: ACCAGCTCTACGCCAAGATG	NM_009504.4
	R: CTTCATGCTGTTCTCCGGCT	
*GAPDH*	F: AACGACCCCTTCATTGAC	M32599.1
	R: TCCACGACATACTCAGCAC	

*AhR*: aryl hydrocarbon receptor; *CYP1A1*: cytochrome P450 1A1; *CYP27B1*: cytochrome P450 27B1; *GAPDH*: glyceraldehyde 3-phosphate dehydrogenase; *IL-22*: interleukin-22; *IL-22Rα*: interleukin-22 receptor alpha; *Muc2*: mucin 2; *Tff3*: trefoil factor 3; *VDR*: vitamin D receptor; F: forward; R: reverse.

## Data Availability

Not applicable.
